# PRESIDENTIAL SYMPOSIUM: BUILDING FORTITUDE FOR THE JOB MARKET

**DOI:** 10.1093/geroni/igae098.1522

**Published:** 2024-12-31

**Authors:** Rita Hu, Sohyun Kim, Marie Bernard

**Affiliations:** Chair; Co-Chair; Discussant

## Abstract

The journey to establish a robust career in the gerontological research field is often marked by navigating through a maze of challenges, strategic decision-making, and the forging of vital professional relationships. The ESPO Presidential Symposium will feature early-career scholars from each scientific section to share their diverse pathways to landing a job and achieving research independence. Dr. Marissa Schafer from Mayo Clinic will discuss transitioning from PhD to Principal Investigator (PI), underlining the necessity of a comprehensive research portfolio, nurturing mentor-mentee relationships, and strategic career moves. Dr. Ketlyne Sol from the University of Michigan will share insights from her clinical psychology path, stressing the importance of dedicated research training and building a supportive network. Dr. Allyn M Bove from the University of Pittsburgh will compare physical therapy principles to career building in research, suggesting a balanced development of strength and endurance. Dr. Sara Bybee from the University of Utah will explore job market strategies through the lens of posttraumatic growth, highlighting personal strength and professional growth opportunities. The symposium concludes with a panel discussion led by Dr. Marie Bernard, the Chief Officer for Scientific Workforce Diversity at NIH, synthesizing the presentations and facilitating a dialogue among the speakers. This symposium serves as a crucial platform for aspiring gerontologists, offering guidance, strategies, and encouragement to navigate the complexities of the job market and achieve a successful and fulfilling career in aging research.

